# Is a clean river fun for all? Recognizing social vulnerability in watershed planning

**DOI:** 10.1371/journal.pone.0196416

**Published:** 2018-05-01

**Authors:** Bethany B. Cutts, Andrew J. Greenlee, Natalie K. Prochaska, Carolina V. Chantrill, Annie B. Contractor, Juliana M. Wilhoit, Nancy Abts, Kaitlyn Hornik

**Affiliations:** 1 Department of Natural Resources and Environmental Sciences, University of Illinois at Urbana-Champaign, Urbana, Illinois, United States of America; 2 Department of Urban and Regional Planning, University of Illinois at Urbana-Champaign, Urbana, Illinois, United States of America; University of Hyogo, JAPAN

## Abstract

Watershed planning can lead to policy innovation and action toward environmental protection. However, groups often suffer from low engagement with communities that experience disparate impacts from flooding and water pollution. This can limit the capacity of watershed efforts to dismantle pernicious forms of social inequality. As a result, the benefits of environmental changes often flow to more empowered residents, short-changing the power of watershed-based planning as a tool to transform ecological, economic, and social relationships. The objectives of this paper are to assess whether the worldview of watershed planning actors are sufficiently attuned to local patterns of social vulnerability and whether locally significant patterns of social vulnerability can be adequately differentiated using conventional data sources. Drawing from 35 in-depth interviews with watershed planners and community stakeholders in the Milwaukee River Basin (WI, USA), we identify five unique definitions of social vulnerability. Watershed planners in our sample articulate a narrower range of social vulnerability definitions than other participants. All five definitions emphasize spatial and demographic characteristics consistent with existing ways of measuring social vulnerability. However, existing measures do not adequately differentiate among the spatio-temporal dynamics used to distinguish definitions. In response, we develop two new social vulnerability measures. The combination of interviews and demographic analyses in this study provides an assessment technique that can help watershed planners (a) understand the limits of their own conceptualization of social vulnerability and (b) acknowledge the importance of place-based vulnerabilities that may otherwise be obscured. We conclude by discussing how our methods can be a useful tool for identifying opportunities to disrupt social vulnerability in a watershed by evaluating how issue frames, outreach messages, and engagement tactics. The approach allows watershed planners to shift their own culture in order to consider socially vulnerable populations comprehensively.

## Introduction

By privileging hydrological boundaries over political ones, watershed planning has the capacity to disrupt ecological degradation. Also known as boundary organizations, brokers, coalitions, and consortiums, watershed planning groups have worked to catalyze voluntary actions from landowners to improve water quality and reduce pollution [1–3]. Watershed planning for Lake Tahoe, for example, created new opportunities for innovation and partnership that restored the clear waters of the lake [4]. The framework for communication was flexible and allowed stakeholders to identify and act on shared values. The results of this and other work demonstrates the capacity of watershed planning to enhance policy learning and improve environmental protection [1, 2, 4–7]. This suggests that the collaboration and cooperation that emerges around watershed planning might offer a potential pathway toward greater local sustainability. First, partnerships often form because the profound complexity of social-ecological-and technical relationships make it difficult to assign responsibility and authority over water management to a single organization [5–8]. Therefore negotiating across lines of accountability and authority can be a good way of addressing collective benefits.

Secondly, many watershed partnerships seek direct citizen participation [6,9–11]. Yet many are plagued by relatively low engagement with communities most affected by flooding and pollution [9]. As with sustainability initiatives in general [12], many watershed planning groups suffer from poor accounting for social equity [13–15]. This is significant because socially vulnerable groups are often disproportionately affected by hazards, most negatively affected by exposure to social and environmental stressors, and least able to adapt to change [16]. Even in watershed planning processes that feature best practices concerning citizen participation, empowered social groups tend to be overrepresented [17]. Thus, whether watershed approaches can become a tool for furthering social equity and social dimensions of sustainability remains open to question [7].

In the United States, individuals with the following characteristics are often overrepresented in watershed-based planning: male sex, middle aged, married, parent of school-age children, homeowner, access to transportation, long-term resident, high level of income and wealth, employed in paid work, and high level of formal education [9]. This is likely to influence the priorities of the group and who benefits from watershed interventions [9,15]. As a result, many policy interventions enacted by watershed planning groups carry disproportionately large cultural shifts among people of color, women, low-income populations, and other groups that are socially vulnerable [11,18]. Uneven participation results in a paradoxical challenge with regard to achieving better representation of socially vulnerable groups–how does a watershed group know which perspectives are missing in the absence of input from those perspectives?

The patterns of underrepresentation suggest that watershed planners would benefit from understanding whether their definition of socially vulnerable groups are artificially constrained, leading to “blind spots” created by their cultural lens [19]. The persistence of such blind spots undermines the credibility of efforts toward diversifying citizen engagement [20]. Because watershed planning groups often lack clear lines of accountability, the potential for their political malleability to perpetuate, exacerbate, or generate injustice and exclusion may be under-recognized [11,21–23]. If, as the literature suggests, watershed planning is dominated by the “usual suspects”, their messages, modes of communication, and social networks are unlikely to reach socially vulnerable groups, however they are defined [24]. External sources of data may provide assessment tools to inform efforts to identify and engage socially vulnerable groups in ways that are sensitive to the lived experience of vulnerability.

One assessment tool that may be useful to improving recognition of social vulnerability within a watershed is the Social Vulnerability Index (SoVI). SoVI originated in hazards research and is widely used in the U.S. because it is constructed from data collected as part of large public surveys such as the decennial census and American Community Survey. Originally formulated by Cutter et al. [25], SoVI provides a credible and widely employed approach to develop relative measure of vulnerability. Over 32 social and economic variables make up the SoVI index [25]. They are collapsed statistically to form components, which can then be interpreted and added together to categorize the relative level of social vulnerability for a given unit. Past applications to watersheds and flood hazards demonstrate that both relative levels of vulnerability and the correlations among variables that comprise the index are important to understanding the distribution and nature of social vulnerability within the watershed [26].

The promise of SoVI as an analytic technique capable of capturing multiple dimensions of social vulnerability is evidenced in its development. There are many demographic variables associated with community-level social vulnerability. The social and economic variables often explain why communities may experience hazards and benefits in a watershed differently. For example, income, employment accessibility, industry mix, age, gender, race, ethnicity, and other special needs indicate locations with larger proportions of residents with characteristics consistent with social marginalization or reduced coping capacity [14] (see S1 Table for a full list of variables and association with social vulnerability). These variables are often correlated across space and may compound relative levels of vulnerability when present together. For example, a unidimensional measure such as median household income or percent of households below the poverty line would not capture related factors such as high proportions of non-English speaking, female headed households below the poverty line. More importantly, unidimensional measures would not be able to capture how these correlated factors may change together over time. Thus, synthesizing a variety of socio-economic variables to construct components of social vulnerability and combining those components to create social vulnerability measures provides a powerful means of uncovering complex patterns of social and demographic change [25,27–30].

There are several common social vulnerability assessments derived from SoVI. Scholars have extended the SoVI to identify many ways that sociodemographic change is likely to both perpetuate or *create* forms of social vulnerability [29,31–34]. Analysis highlighting temporal dimensions of the SoVI, for instance, have revealed new insight into the cumulative effects of flood hazards on social and economic conditions [26]. SoVI analysis has been downscaled to smaller geographies (such as census tracts) and applied to spatial extents ranging from the entire US to states, regions, coastlines, or other smaller administrative geographies (e.g. [35–39]). SoVI has also proven to be temporally robust and its construction has evolved in response to changes in theory and to technical challenges including changes in the enumeration procedures of the U.S. Census and American Community Survey [40].

If local definitions of social vulnerability are generally consistent with the variables that make up the SoVI, we anticipate that the index will provide an analytical framework with sufficient flexibility to differentiate and delineate the geographies of multiple forms of social vulnerability within a watershed. At the same time, efforts to understand the extent that socially vulnerable groups have been adequately represented in watershed planning provides a unique opportunity to contribute to the literature surrounding the index itself. SoVI has been critiqued for being insufficiently grounded in lived experiences and political and economic context (e.g. [41,42]. In response to this critique, researchers are beginning to engage stakeholders in the process of index validation, weighting, and revision (e.g. [41,42]).

To advance efforts toward more equitable and inclusive watershed planning, this paper tests three hypotheses. First, we test the hypothesis that social vulnerability within a watershed carries multiple operational definitions. Second, we test the hypothesis that watershed planners have an incomplete understanding of the dimensions of social vulnerability that are relevant to their work. Third, we test whether geospatial techniques used to assess social vulnerability can be modified to recognize diverse forms of social vulnerability within a watershed. The rationale for this hypothesis is that social vulnerability exists at the intersection of social inequalities and place inequalities [43]. As an intersectional phenomenon, social vulnerability is likely to be expressed in several different ways—if not adequately recognized and represented, then watershed planning may perpetuate or exacerbate the normalization and naturalization of an exclusionary environmental worldview [19]. We develop two studies to test these hypotheses. The first analyzes interviews to generate stakeholder perceptions of social vulnerability and the adequacy of watershed planner perceptions. The second study aims to construct spatial representations of vulnerable census tracts in correspondence with each definition. We us the Milwaukee River Basin, which includes much of the highly segregated city of Milwaukee, Wisconsin (USA) as a study site because of its demonstrated need to overcome a history of exploited waterways and high social vulnerability [44]. The result is a tool aimed at improving efforts to transform ecological, economic, and social relationships through watershed planning. Examining the suitability of the SoVI as an assessment tool in light of interview responses contributes to the literature through its test the robustness of the index itself and by uncovering the limits to inclusion.

### Watershed planning in the Milwaukee River Basin

We test our hypotheses in the Milwaukee River Basin watershed. This is an ideal case study because planning efforts span over 35 years and have included major interventions such as large investments in sediment remediation [45,46]. The contribution of the Milwaukee River Basin to the ecological integrity of the Great Lakes [46] and the recent finding that many watershed management groups in the Midwestern US lack adequate mechanisms for inclusive stakeholder engagement [11] make it a case of significance nationally, if not globally. The Milwaukee River Basin drains into Lake Michigan, one of five Great Lakes at the border of the US and Canada. The Great Lakes hold 20 percent of the surface freshwater on the earth. The Milwaukee River Watershed contains around 1.3 million people, spans portions of seven counties, and contains 13 cities, 32 towns, and 24 villages. The watershed ends in the city of Milwaukee, and includes the Menomonee, and Kinnickinnic Rivers, which also traverse the city of Milwaukee [46]. Known as the “Selma of the North” Milwaukee has a long history of social segregation that is intimately tied to a reliance on waterways as lines of separation emphasized by their use as a means of transporting waste [47].

The inception and evolution of watershed planning in the Milwaukee River Watershed has improved ecological function and regional economic opportunities [48]. However, efforts to engage with pernicious social issues are relatively new given more than three decades of planning intervention. Concerted watershed planning efforts in the Milwaukee River Basin began in 1979 with the adoption of the Wisconsin Regional Water Quality Management Plan. Although not the first watershed-based planning initiative within the region, the plan (and its subsequent updates) maintains a singular focus on natural systems pollution [49]. In the years that followed, significant federal incentives for watershed-level planning and changes in natural resource planning at the state level facilitated the formation of the Milwaukee River Basin Land and Waters Partner Team in 1998 [50]—one of eighteen watershed planning units established in Wisconsin [45].

By 2007, a complimentary economic development initiative, the Milwaukee River Water Council was formed, lending greater institutional weight to the watershed as a governance and planning unit [51]. Concurrent with the formation of the Milwaukee River Water Council, the City of Milwaukee developed plans to become a hub for water research, economic development, and education. Taken together, these plans support coordination around flood risk, groundwater depletion, pollution, habitat degradation, and reduced recreational opportunities [50,52].

Today, the Milwaukee River Basin Land and Waters Partner Team coordinates watershed planning. The group is a "voluntary coalition committed to restoring & sustaining the Milwaukee River Basin ecosystem, while ensuring economic viability. The partnership promotes comprehensive resource management, information exchange, intergovernmental coordination, & citizen involvement" [53]. It is an important integrating unit between environmental cleanup initiatives in the watershed undertaken through federal remediation programs designed to remediate contaminated tributaries to the Great Lakes, local restoration and water quality improvement efforts, and statewide efforts to reduce sources of water pollution from both agricultural and urban sources [50].

## Study 1. Local definitions of social vulnerability

Study one analyzes interviews with stakeholders in the Milwaukee River Basin in order to test the hypothesis that social vulnerability within a watershed carries multiple operational definitions and that watershed planners have an incomplete understanding of the dimensions of social vulnerability.

### Methods

We conducted 31 semi-structured interviews with 35 stakeholders in the Milwaukee River watershed (Table 1). The purposive sampling frame selected participants to capture variation across a gradient experience in water management and social knowledge through formal means (e.g. as government or non-governmental organization (NGO) employees) or informal ones (as a resident). Two groups participated in watershed management (government officials and environmental NGOs) and three did not (community NGOs focused on social equity, influential community leaders, and residents of the watershed with no relevant professional interest). Interview questions elicited definitions of social vulnerability by asking “How would you describe the social groups that are most vulnerable in relation to the environment?” and asked probing questions to encourage participants to substantiate why they identified particular people and places. The study was reviewed and approved by the Institutional Review Board at the University of Illinois (approval number 14431). Informed consent was obtained from participants in writing before the interview portion of the research as specified in the protocol. Among participants, males (n = 23) were better represented than females (n = 12). We did not ask respondents to share their personal racial and ethnic identity.

**Table 1:** Interview classification and definitions of social vulnerability.

	Definition of social vulnerability					
Stakeholder group	Spatial	Temporal	Persistent	Increasing	Transient	Total
*involved in watershed planning*						
government official	1	-	-	2	2	6
environmental NGO	3	-	-	1	-	4
*not involved*						
community NGO	-	3	4	-	4	11
community leader	1	1	-	-	2	4
resident	2	1	3	1	-	6
**Total**	**7**	**5**	**7**	**4**	**8**	**31**

We used open coding strategies to analyze responses and identify definitions of social vulnerability, the details of which appear elsewhere [54]. Over several iterations of theme identification and validation across investigators, we identified 5 distinct conceptual definitions of social vulnerability, which varied based upon the extent to which stakeholders weighted socioeconomic and demographic factors, geography, and historical context in their descriptions (Table 1).

Stakeholder framing of temporal and spatial change emerged as a primary factor that differentiated among five definitions of social vulnerability. Of these, water managers expressed concern for spatial, increasing, and transient forms of social vulnerability, omitting two other forms defined in the larger set of interviews.

### Results

#### Definition 1: Spatial social vulnerability

The first definition of social vulnerability focuses on spatially identifiable forms of vulnerability, and does not consider historical factors or change over time as important to identifying social vulnerability. For these respondents, vulnerability was “definitely race and socioeconomics”, and vulnerable communities are clearly defined by geography. As one interviewee stated:

Milwaukee is pretty easily divided, very segregated. So it’s like the northeast corner is white people with money. They’re either students or rich, working professionals. The northwest side of the community is where the black people live. The southwest is where the Mexican people live, and then the southeast is where young, white families have houses in Bay View, and it’s kind of a little bit of mix of everybody–Resident, Interview 27

The spatial definition of social vulnerability was expressed broadly across stakeholder groups (Table 1).

#### Definition 2: Temporal social vulnerability

In interviews, a second definition of social vulnerability focused on demographic change and transition without a specific emphasis on geographic location. Respondents focused on the emergence of new ethnic groups, like the Hmong, who might be more vulnerable because of cultural differences or language barriers as they navigated through the world. This vulnerability was expressed by respondents engaged in watershed planning. As one said:

And there were a lot of opportunities, in the sixties and seventies, the workforce became more diverse, the workplaces were full of people of all races and genders, African Americans and women were breaking barriers in terms of getting higher paid jobs in the skill trades and professional type situations, but now with the depressed economy it makes it that much harder for people who are looking to break barriers, it makes it harder in difficult economic times to break barriers, don’t you think?–Environmental NGO, Interview 17

This perspective was widely shared among interview participants with high social knowledge, but was not used by any of the interviewees participating in watershed planning (Table 1).

#### Definition 3: Persistent social vulnerability

For participants using the persistent vulnerability definition, power relationships keep communities vulnerable. Therefore, efforts to find solutions often come from external sources. The ultimate result is that knowledge is extracted from the community without any expectation of reciprocal investments. This process divides and disengages the community from itself, allowing it to become further disempowered. Respondents focused on a lack of agency and participation that allowed social vulnerability to be a constant concern (Table 1). One participant commented:

Who is invited to the table? Who even gets to decide who sits at the table? It's not like they're seen and it's like, "Oh, I won't go a meeting." There's no expectation that there's any reason to participate in those kinds of things.–Resident, Interview 29

As with temporal vulnerability above, this was a perspective did not emerge in any interviews with watershed planners.

#### Definition 4: Increasing vulnerability due to a more deeply divided society

The fourth definition of vulnerability is concerned with increases in social vulnerability over time. It is similar to persistent vulnerability, except that it is more explicit about historical patterns of racism, classism, and sexism. The definition emphasizes the ways that the industrial legacy of Milwaukee has led to vulnerability traps in some neighborhoods, by which neighborhoods become increasingly vulnerable to the ill effects of both negative and largely positive environmental work. Interviewees said things like:

…In my experience, [social disparity across race and ethnicity] either–it's probably hovered between maybe stagnating and worsening, I think. And I think that it's tied to mostly, again, this whole opportunity–jobs element, and it's very challenging. There's a lot going on education-wise and challenges, as well as with the school systems in the inner city. That has continually been a major source of controversy and a lot of effort–try to remediate that.–Government official, Interview 23

This definition of social vulnerability was least common overall, but was expressed by both to watershed planners and other stakeholders (Table 1).

#### Definition 5: Transience as a form of social vulnerability imbued on a place

The last definition of social vulnerability is defined by high population turnover and was expressed by both watershed planners and others (Table 1). For respondents using this definition of social vulnerability, the transience of the population limits social capital and place attachment and, therefore, the ability of the neighborhood to form a cohesive identity. Participants identified social vulnerability as equivalent to low institutional memory and say this as relevant for regions in which high population turnover had reduced political engagement and therefore the capacity of resident interests to be included in future decisions. As one interviewee stated:

Even in quality of life and green space, as areas get built up and gentrification might set in and people get priced out of their homes and their living spaces and they again don’t get to live in this area that maybe has a lot more beautiful green spaces. So it’s these outside forces that are maybe creating some great change for the environment, but then those folks don’t get to enjoy it. Now if they have a leadership role and can help make or change or discuss some of the ordinances surrounding that development and they’re protected, they’re going to have a lot stronger quality of life.–Community NGO, Interview 13

## Study 2: Recognizing and locating social vulnerability using geospatial models

The second study tests whether geospatial techniques used to assess social vulnerability can be modified to recognize diverse forms of social vulnerability within a watershed. We examined whether the existing SoVI offers a sufficiently flexible framework for assessment or multiple definitions of social vulnerability. After verifying that the underlying conceptual assumptions of SoVI construction were amenable to the varying definitions of social vulnerability expressed in interviews.

### Methods

**Fig 1:**
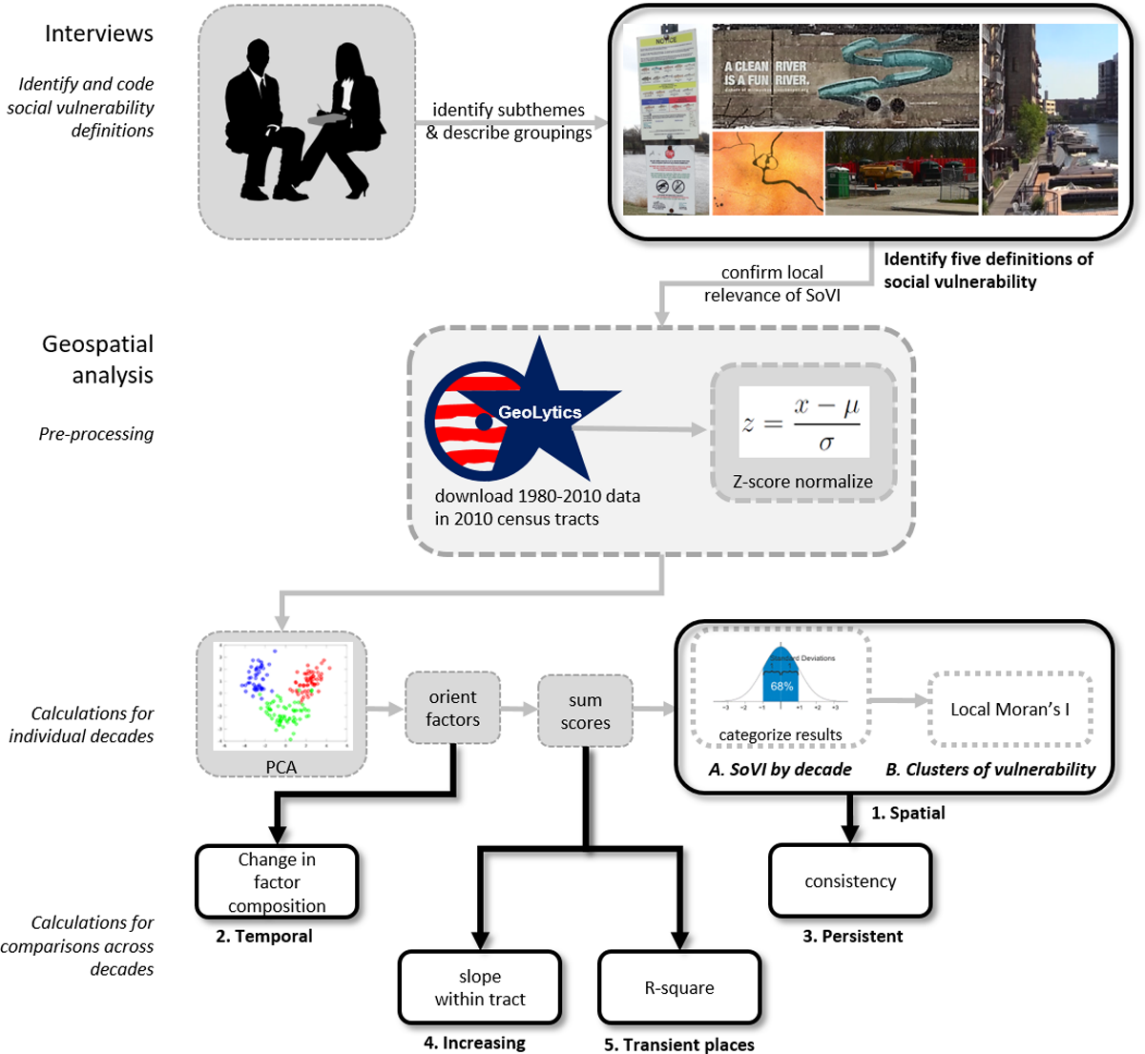
Flow diagram indicating methodological framework and resulting measures of social vulnerability. Gray arrows and boxes show intermediate data processing steps. Black arrows and boxes connote results.

This methods sections outlines the protocol for quantifying and visualizing alternative forms of social vulnerability expressed in interviews. To evaluate the sufficiency of SoVI with respect to community definitions, we assessed whether social and demographic variables comprising the index were also mentioned in interview responses. Then, we checked the neighborhood boundaries that were mentioned specifically within our interviews to confirm that census tracts were an acceptable minimum areal unit. Once confirmed, we constructed separate measures for each of the expressed definitions of social vulnerability (Fig 1). This section provides the rationale and analytic techniques used to match each social vulnerability definition to empirical assessment. This resulted in the development of two novel assessment techniques designed to characterize increasing vulnerability over time and vulnerability due to relatively high levels of transience. To our knowledge, neither of these measures have appeared in the literature previously.

To accommodate temporal dimensions of the social vulnerability definitions offered by stakeholders, we drew from census data across multiple decades. We elected to include decades that bracketed the emergence of watershed-based planning and to standardize boundaries and data sources across decades to allow for direct comparison. We assembled longitudinal tract data for the years 1980, 1990, 2000, and 2010, using decennial census data and data from the 2006–2010 American Community Survey (which supplements “long form” questions omitted from the 2010 Census). We followed best practices to reduce index sensitivity to data availability associated with the changes in the 2010 Census and American Community Survey [55].

To accommodate changes in census tract boundaries across decades, we used data from the Geolytics Neighborhood Change Database [56], which reapportions historical census data into 2010 census tract boundaries. This resulted in the loss of seven variables from the 32 variables used by Cutter et al. [28] at the census tract level. These variables were: (1) number of hospitals per capita, (2) median age, (3) percent change in foreign born population, (4) average number of people per household unit, (5) number of people per 100,000 employed as healthcare practitioners and technical occupations (6) percent rural farm population (7) percent urban population. The loss of these variables did not substantially alter vulnerability calculations. Comparisons between index scores for particular decades in which the variables were present in original US Census data (but not in the Neighborhood Change Database developed by Geolytics), indicated that the variable loss was not influential in the construction of the social vulnerability index for the Milwaukee River Basin or its component (authors, unpublished data). The remaining 25 variables were included in index construction based on their theoretical influence on social vulnerability and consistency with the definitions of social vulnerability offered in interviews (Table 2).

**Table 2:** Descriptions for the 25 input variables considered in the social vulnerability index (SoVI). Adapted from [28].

Variable	Description	Relationship to social vulnerability
HODENT	Number of housing units per square mile	+
M_C_RENT	Median contract rent	-
MHSEVAL	Average owner occupied home value	-
NRREPC	Per capita residents in nursing home	+
PCTRICH	% of FAMILIES earning $100,000 +	-
PERCAP	Per capita income (dollars)	-
QAGRI	% employed in farming, fishing, and forestry	+
QASIAN	% Asian & Pacific Islander	+
QBLACK	% African American	+
QCVLBR	% of population participating in the labor force	+
QCVLUN	Unemployment	-
QED12LES	% of population 25+ with no high school diploma	+
QFEMALE	% female population	+
QFEMLBR	% of women participating in the labor force	-
QFHH	Female headed families & sub-families with children	+
QINDIAN	% Native American	+
QKIDS	% population > age 5	+
QMOHO	% mobile homes	+
QPOP65	% of population age 65+	+
QPOVTY	% of population below the poverty line	+
QRENTER	% renter occupied housing	+
QSERV	% employed in service industry	+
QSPANISH	% Hispanic	+
QSSBEN	% of households collecting social security	+
QTRAN	Employed in transportation, communication, and other public utilities	-

### Results

#### 1: Spatial social vulnerability (SoVI using watershed boundaries and cluster analysis)

Assessing spatial vulnerability required a spatially referenced (and temporally static) measure of social vulnerability, but were unable to know whether the aggregation of vulnerability into larger clusters was important. As a result, we calculated two alternative measures, both of which have appeared in the literature previously. We did this by constructing a vulnerability index for each decade within the watershed boundaries [31,32,57]. This reflects the traditional SoVI indexing strategy used to represent social vulnerability applied to each decade individually following the procedure outlined below. We followed this with a measure of clustering (described in the section below).

To express the data as a reduced set of uncorrelated components for each decade, we perform Z-score transformations on each variable and completed principle components analysis (PCA) (Fig 1). Z-score transformations standardize variables by centering values rescaling the distribution to a common range. The standardization and rescaling of variables is a typical first step within both inductive and deductive approaches to social vulnerability indexing, as it creates a uniform range of variance across indicator variables, an important precondition for this implementation of indexing with PCA. PCA is an inductive data reduction approach to social vulnerability indexing [30,58]. First, component scores are assigned a direction so that higher scores indicate high levels of social vulnerability. This allows for the construction of an aggregate index where components that increase vulnerability have a positive influence on index scores while those that decrease vulnerability have a negative influence (Table 2). Next, all components scores are added together for individual census tracts and the distribution is normalized by Z-score (like [14]). Varimax rotation minimizes the association between individual variables to multiple components [59]. We retain only components with an eigenvalue greater than one.

**Fig 2:**
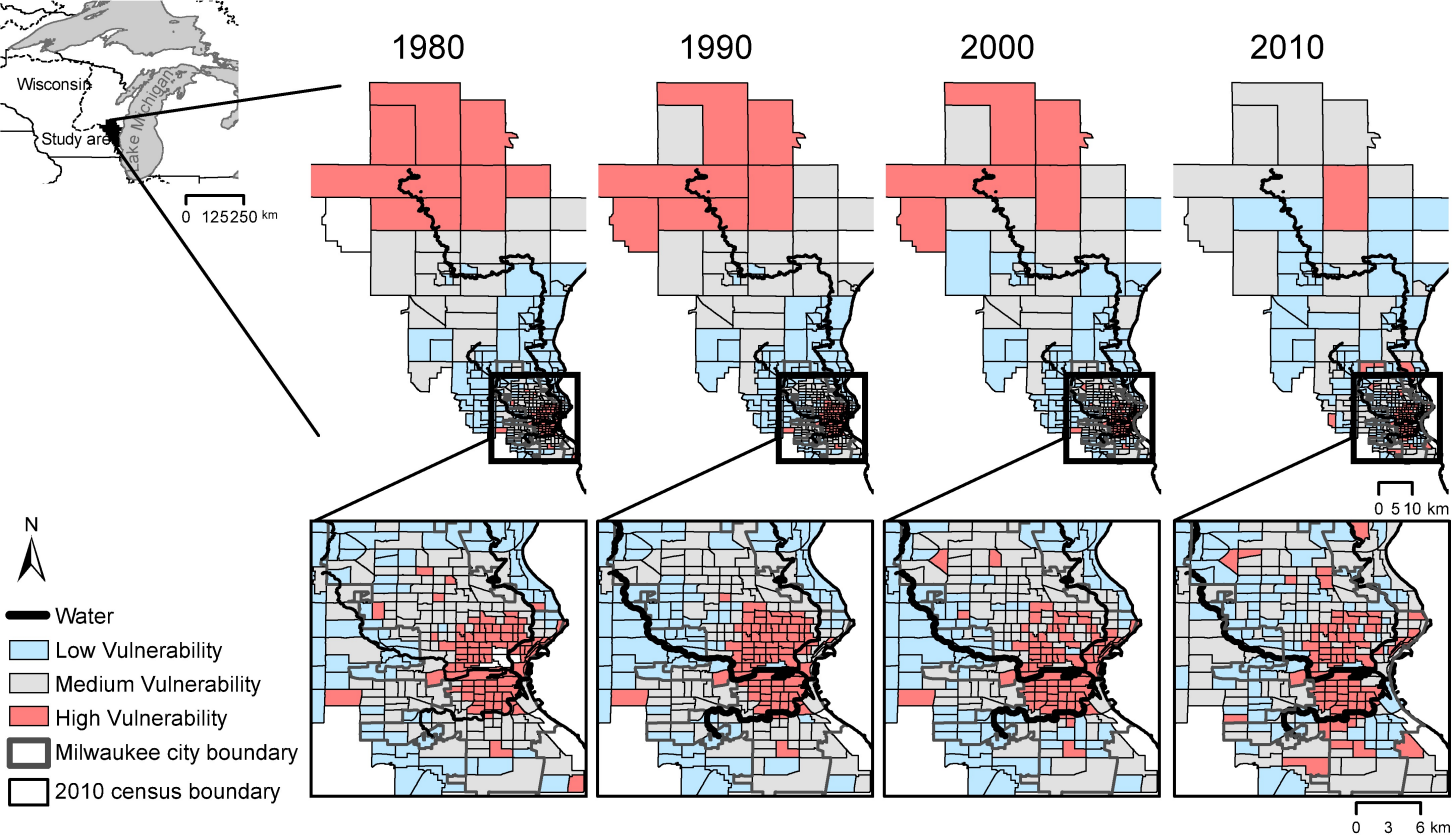
Tract-level social vulnerability index (SoVI) results for the Milwaukee River Basin from 1980–2010. Inset map locates the Milwaukee River Basin in reference to Lake Michigan and the Great Lakes along the border of the USA and Canada. The upper panel show trends for the watershed with dark red indicating the highest level of social vulnerability for census tracts in 1980, 1990, 2000, and 2010. The lower panel shows the same data within the city of Milwaukee. Data for all maps is reapportioned to 2010 census boundaries in the Geolytics Neighborhood Change database.

PCA resulted in six-component solutions for 1980 through 2000, and a seven-component solution for 2010. Each solution explained between 71 percent and 76 percent of variation in the underlying data. In all census years, the first component explained more than 34 percent of the variance in the data. Table 3 provides a summary of these components including our interpretations of each. Fig 2 maps aggregate social vulnerability for each period (ordered categories of vulnerability rankings are divided into three categories using ± 0.5 standard deviations from the mean to differentiate high, medium, and low vulnerability categories).

**Table 3:** Measure used to operationalize the temporal definition of social vulnerability. Components (C) elicited for each decade. Component names connote the attributes with theoretical links to high vulnerability. The directional effect (DE) indicates whether the initial component scores needed to be reversed so that higher values were associated with greater vulnerability. The percent of the variance in the underlying data explained by each component is also provided. Full PCA results are provided in S1 Table.

C	1980	DE	% of variance	1990	DE	% of variance	2000	DE	% of variance	2010	DE	% of variance
C1	Low income, African-American communities	(+)	37.02	Low income, African-American communities	(+)	40.71	Low income, African-American communities	(+)	40.99	Low income, African-American communities	(+)	34.08
C2	Advanced age dependents	(-)	13.39	Low participation in labor force	(+)	9.95	Fixed income senior citizens*	(-)	10.07	Low participation in labor force	(+)	11.25
C3	Low income, non-African American communities	(-)	8.65	Low income, non-African American communities	(-)	8.30	Hispanic and/or Native American communities	(+)	8.83	Hispanic and/or Native American communities	(+)	8.71
C4	Low participation in labor force	(-)	7.01	Hispanic and/or Native American communities	(+)	7.40	Advanced age dependents	(+)	7.11	Advanced age dependents	(+)	6.17
C5	Hispanic and/or Native American communities	(+)	5.35	Low income Asian communities	(-)	6.27	Low participation in labor force	(+)	6.00	Low income Asian communities	(+)	5.47
C6	Low income, low stability communities	(+)	4.32	Low income, low stability communities	(+)	4.12	Low income, low stability communities	(+)	4.38	Low income, female headed households	(+)	4.66
C7	n/a	n/a	n/a	n/a	n/a	n/a	n/a	n/a	n/a	Low income, low stability communities	(-)	4.35
**Total**			**75.75**			**76.75**			**77.38**			**70.34**

Note: * fixed income seniors are part of “Advanced aged dependent” in 1980, 2010

**Fig 3:**
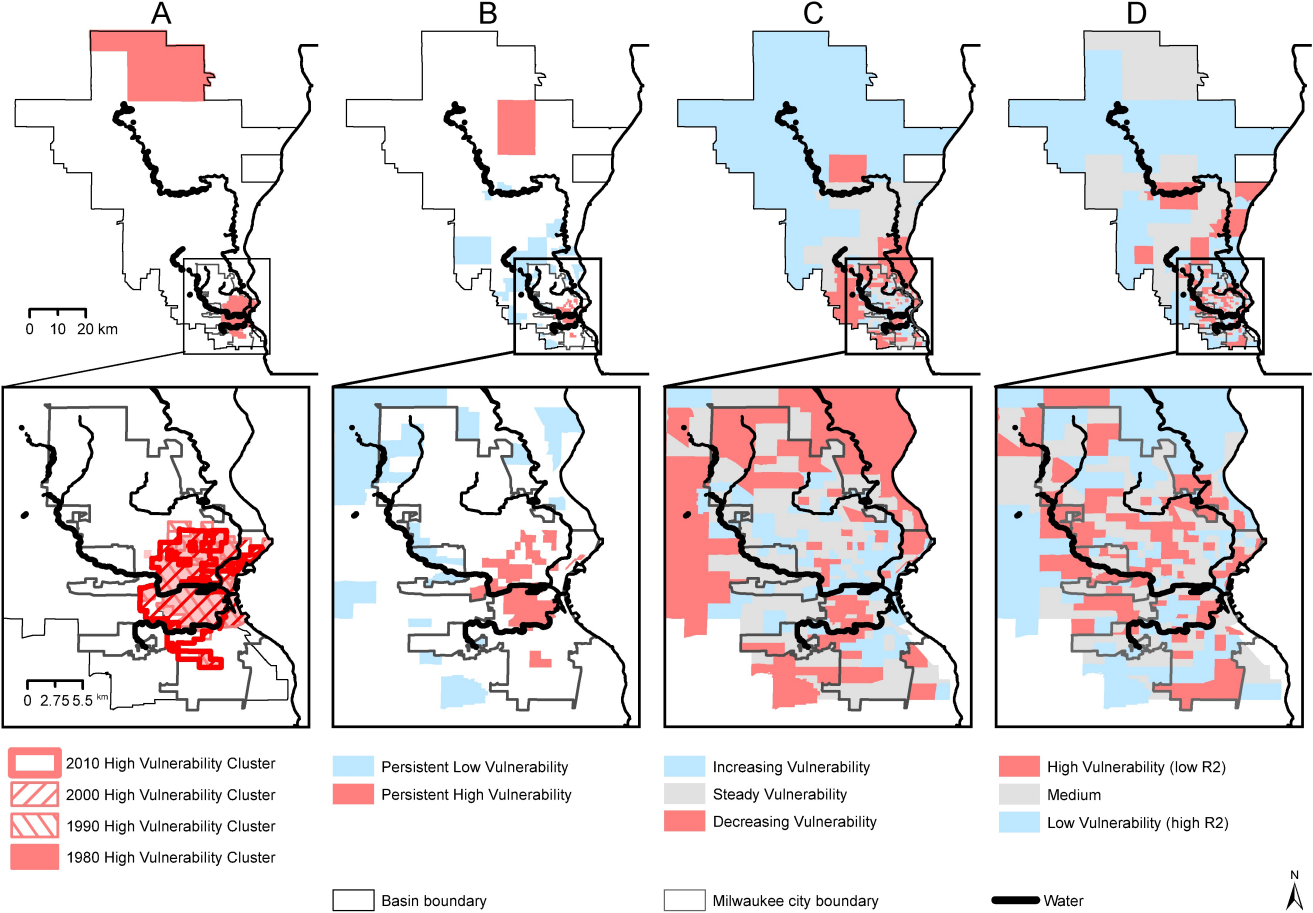
Maps of measures of vulnerability over time corresponding to Table 4. Clusters of high vulnerability for 1980, 1990, 2000, and 2010 appear on the far left. With persistent vulnerability, increasing vulnerability, and transient vulnerability following.

The literature suggests a second measure able to assess vulnerability under the spatial definition. This measure focused on spatial clusters of social vulnerability, or areas where census tracts with high vulnerability are statistically more likely to be located near other high vulnerability tracts [31]. To identify clusters, we calculated Global Moran's I to test for spatial autocorrelation, or a non-random distribution of social vulnerability scores. A positive value of the Moran’s I indicates high level of clustering. A negative Moran’s I indicates tendency towards dispersion. We find that social vulnerability index scores are clustered through the watershed (Global Moran’s I scores were 0.23; 0.25; 0.26; and 0.16 for each decade (p<0.001) indicating a tendency for census to have similar vulnerability values to their neighbors). To identify high vulnerability clusters in each decade, we used Local Moran's I, which measures the relative similarity of neighboring locations [60]. In this study, spatial clusters of high social vulnerability are census tracts with high aggregate vulnerability (Measure 1-A) surrounded by features with similarly high values. For each decennial year, there are between two and four high vulnerability clusters (Table 4). These clusters are comprised of between 80 and 85 census tracts in 1980, 1990, and 2000. In 2010 fewer high vulnerability tracts were associated with clusters, suggesting some dispersion of vulnerability (Table 4). Vulnerability clusters close to the waterfront change over the study period and are more diffuse by 2010 (Fig 3A).

**Table 4:** Descriptive table of social of vulnerability measures corresponding to maps in Fig 2.

Concept	Description	Min	Max	# of Tracts	% of Tracts
A. Clusters of (high) vulnerability	1980*(4 clusters)	n/a	n/a	83	0.25
	1990 (2 clusters)	n/a	n/a	82	0.24
	2000 (2 clusters)	n/a	n/a	85	0.25
	2010 (4 clusters)	n/a	n/a	72	0.21
B. Persistent Vulnerability (stable assignment to the same vulnerability class across all decades)*	Persistently High Vulnerability	n/a	n/a	45	0.13
	Persistently Low Vulnerability	n/a	n/a	43	0.13
C. Increasing Vulnerability (slope of line fit through vulnerability scores from 1980 to 2010)*	Decreasing vulnerability (≤-½σ)	-2.33	-0.17	67	0.20
	Steady vulnerability (-½σ<μ<½σ)	-0.17	0.15	202	0.60
	Increasing vulnerability (≥½σ)	0.15	1.07	68	0.20
D. Vulnerability of transient places (R-square of a line fit through vulnerability scores from 1980 to 2010)*	High vulnerability due to high churning (≤-½σ)	0.00	0.34	100	0.30
	Medium churning (-½σ<μ<½σ)	0.34	0.70	115	0.34
	Low churning (≥½σ)	0.70	1.00	122	0.36

* there are 5 tracts with null values of SoVI due to null values in some variables in 1980.

#### 2: Temporal social vulnerability (changes in SoVI components across decades)

To assess the temporal definition of social vulnerability, we evaluated the relationships among variables changes between the social vulnerability index calculation for each decade [31,61]. The changes in component structure and explanatory power over time provide insight into how the social configuration of the Milwaukee River Watershed and, therefore, social vulnerability might have shifted. This highlights changes in intersectional identities at the community level, which is consistent with data in the interviews. The component with the most consistent form addresses vulnerability in low-income African-American populations (Table 3). It is comprised of the same variables over time (see online supplementary material, S2–S5 Tables). The variables comprising other components are more dynamic across years and we note two major transitions that occur over the four decadal censuses. First, the variable female headed households loads onto a unique component in the year 2010 (low income, female headed households) when compared to the prior three censuses, where it is part of the first component ('low income African-American communities') and a component named 'low participation in labor force' (Component 4 in 1980; 2 in 1990; and 5 in 2000). Second, the variance in the data attributable to change in Hispanic and Native American households rises over time, explaining 5 percent of variance in 1980 and nearly 9 percent by 2010 (Table 3).

#### 3: Persistent vulnerability (high vulnerability across time)

To capture persistent forms of social vulnerability in the watershed, we constructed a novel measure of vulnerability based on whether measures of social vulnerability were persistently high or persistently low across all decades (see Fig 3B). Following earlier approaches [32,57], we identified 45 census tracts with persistently high social vulnerability across all four decades of our analysis. These tracts are those that stayed in the “high vulnerability” category over all measurement periods (had aggregate social vulnerability scores that were greater than + 0.5 SD from mean vulnerability score over all periods). All but one of the tracts are within Milwaukee city boundaries. In contrast, most of the 43 tracts we identified as having persistently low vulnerability are located outside the municipal boundary.

#### 4: Increasing vulnerability (vulnerability trajectories)

To identify areas of increasing social vulnerability, we constructed a line of best-fit through scores for 1980, 1990, 2000, and 2010 using linear regression through the normalized social vulnerability scores calculated for each decade (before they were assigned to high, medium, and low categories). By observing the slope of each line and comparing it to the slopes of lines for all other census tracts, we assess trajectories of change. As before, we created ordered categories of relative vulnerability change by Z-score normalizing the calculated slopes and classifying them by using ± 0.5 standard deviations from the mean. The results highlight 49 census tracts for which vulnerability is increasing. Conversely, 41 tracts have slopes reflecting decreasing vulnerability (Fig 3C).

#### 5: Transience (high place vulnerability due to demographic turnover)

To assess transient social vulnerability, we construct a new measure of social vulnerability using the R-squared values calculated for each line of best fit. The measure assesses the degree of demographic churning present in the tract (Fig 3D). Across all tracts, the mean R-square is 0.54. We identified 100 census tracts experiencing volatility across our study period. Volatility in this case represents the lowest R-square values using ± 0.5 standard deviations to classify into categories.

### Discussion

The results of this study suggest that social vulnerability is not a monolithic concept. It has many different operational definitions at a watershed scale. Moreover, watershed planners do not have a comprehensive understanding of the ways that social vulnerability operates within a watershed. This supports the results of other studies, which find that watershed planning groups rarely address social inequity [9,17]. Better recognition and inclusion of the perspectives of socially vulnerable populations is not merely an issue of improving biophysical outcomes or how they accrue to beneficiaries, but of reconfiguring how the experience of social vulnerability are understood and recognized in the course of inviting participation before issues are even framed.

The finding that watershed planners have an incomplete conception of social vulnerability demonstrates a need for methods that that provide insight into how and why efforts toward inclusion and broad participation fall short. To this end, the SoVI assessment technique may offer a useful tool to help watershed groups understand the limits of their own conceptualization of social vulnerability and can be used as part of efforts to invite broad and meaningful engagement. Across all definitions of social vulnerability, change over time was an especially relevant feature. Social Vulnerability measures 1-A, 1-B, and 2 (Fig 1) apply social vulnerability concepts developed by environmental hazards researchers to a watershed planning context. Changes in variable associations across decades in Measure 2 highlights changes in demographic patterns that have occurred since 1980 when many of the watershed-based initiatives in the Milwaukee River basin began. These changes may be masked by analyses that rely solely on racial or economic indicators to represent vulnerability. Thus, a multidimensional analysis approach helps to reveal flaws or blind spots in prior plans and interventions that may need to be reevaluated to better accommodate evolving social relationships within the watershed.

Our analysis of the temporal dimensions of change (Table 3) reveal several noteworthy points. Our component interpretations highlight the role of race and ethnicity, income, labor force participation, and age in structuring vulnerability. Race and ethnicity, in particular, are important factors—an observation which conforms with the findings of other studies of social vulnerability (see for instance [62,63]). First, the primary component loading across all four years is low-income African-American communities—the consistency of this component conforms to the historical role which race has played in mediating opportunity and neighborhood stability in Milwaukee and other rust belt cities [64]. While racial dynamics in Milwaukee have arguably changed since 1980, the relative sociodemographic conditions for low-income African Americans remain largely unchanged over the three decade study period. Looking down the component loadings, the intersection income, race and ethnicity, and age also consistently explain large proportions of the data’s variance. In the year 2000, Fixed-income senior citizens emerge as a separate category from advanced age dependents. This may be a separate cohort effect associated with older workers retiring or facing the prospect of a shrinking labor market in the Milwaukee Watershed over this decade. By 2010, the intersection of gender and income emerges as a distinct factor. Literature from sociology has pointed to heightened levels of vulnerability for low-income female headed households in rural areas [65,66], but also to particular vulnerabilities for urban-dwellers [67–69].

The presence of statistically significant clusters of high vulnerability (Fig 3) provides evidence for the presence of an intermediate scale that may simultaneously mediate neighborhood-watershed relations while also requiring substantial multi-stakeholder collaboration across jurisdictions.

Measures developed to address increasing social vulnerability and transient places represent novel contributions to the development and theorization of social vulnerability more generally. These measures address social vulnerabilities that may be unique to watershed-based planning. Whereas increasing social vulnerability is relatively straight forward, transience shifts the vulnerability focus from the people to the place.

The observation that demographic churning erodes political representation is supported by empirical analysis across rust belt cities in the U.S. Although most neighborhoods remain demographically stable [70] changes akin to those captured by our transience measure have been observed [71–72]. While our transience measure does not explain the ways in which these factors drive change, it does help to identify where demographic instability is occurring in order for watershed planners and other groups think about how they intervene in the complex social, economic, and governance factors behind such change. For watershed planners, the transience measure may be particularly important when coupled with other measures of sociodemographic or environmental change. The identification of demographic churning offers the potential to think more closely about the contribution of watershed interventions and other related governance processes to the broader suite of local change dynamics. Taken with other measures of vulnerability, the transience measure can also help planners to think critically about the direction of demographic change and the ways in which narratives associated with these changes can help to identify differential impacts by race, income, and gender. Identifying such zones of (potential) under-representation in watershed planning can thereby provide the basis for improving outreach efforts designed to reach stakeholders and anticipate challenges to adequate problem identification and inclusive participation.

By eliciting definitions of social vulnerability from watershed institutional stakeholders and comparing their conceptual models with social vulnerability indicators, we are able to assess the applicability of these measures as an information source regarding structural shifts driving social inequity. Simultaneously, the results contribute to a growing body of literature that seeks to “ground truth” social vulnerability measures by comparing and contrasting them with the experiences of local residents and decision-makers (e.g. [41,42]).

Across interview participants, definitions of social vulnerability varied, with current watershed planning participants falling short of the full set of definitions in operation.

By dimensioning which vulnerability factors tend to work in concert with each other (and how they have changed over time), the results from this paper can better account for political and economic relationships within planning interventions. As researchers have continued to investigate the utility of social vulnerability metrics, there has been a growing need to attend to context in order to construct better conclusions about the importance of different dimensions of social vulnerability or the extent of their influence over particular hazards [26].

As a result, our study bridges that gap by informing index construction with particular definitions of social vulnerability expressed by interview participants. We deconstruct social vulnerability and include important geographic and temporal characteristics. As a result, our analytics respond to calls for more temporal measures of social vulnerability [33,73] and for better integration with relevant stakeholders [41].

Including and representing socially vulnerable populations is critical to realizing the dual goals of improving ecology and ameliorating injustices in water resource sustainability efforts [23,74,75]. As watershed groups aim to be more inclusive, the social vulnerability measures presented here may provide a useful device for broadening awareness of the ways that their vision for who benefits from environmental projects and how to identify and engage communities that experience vulnerability in different forms. Plans to protect the natural world may advocate for disproportionately burdensome cultural shifts among people of color, women, and low-income populations and other groups that are socially vulnerable [18]. To tackle this deficit, watershed planning groups must address the possibility that their plans are built around assumptions that ignore or reproduce social inequity.

In order to understand whether the social vulnerability assessments suggested here can change the nature of watershed planning in order to enhance concern for social justice, future work should assess outcomes in relation to the application and integration of such analyses within planning processes. For example, Fig 3B and 3C show rapid changes in vulnerability scores in ten tracts. These are contiguous to the Milwaukee Riverwalk, an area of significant investment in the waterfront as an amenity. While this is promising from environmental and economic perspectives, planners must recognize that rapid changes in vulnerability scores may also be reflective of residential displacement—a phenomenon that would likely result in increased vulnerability for the neighborhood itself as well as for the residents of households that relocate.

### Conclusions

This paper represents a departure from the more common “deficit model” in which the implied “wrong” is among those not participating. By arguing that the worldview of the watershed planners might itself be incomplete, we turn the tables, encouraging watershed groups to rethink how and where they identify community interests and partners. Our approach can help identify and resolve challenges to creating a just sustainability in urbanized watersheds. Researching the social context of environmental interventions is an important to deeper recognition of the interdependence of social and ecological systems. We draw heavily from the literature on the social vulnerability index and its alternatives, as they have been developed in the field of hazards geography.

The capacity for watershed-based ecological improvements to enhance environmental justice is limited without a broad understanding of social vulnerability. Each vulnerability measure suggests a different planning challenge (Table 5). Environmental improvements have the potential to be a double-edged sword, enhancing environmental quality while also enabling displacement that exacerbates vulnerability. Projects like the Milwaukee Riverwalk have experienced substantial public and private investment [76]. Funding may address immediate deficits in terms of structural problems, but more sustained support will be necessary to achieve long-term environmental protection once a specific project ends [77].

**Table 5:** Summary of measures of vulnerability developed in this paper and their uses to inform planning.

Measure	Method	Contribution to Inclusionary Planning
1. Spatial Vulnerability	1-A. Description of aggregate social vulnerability scores across space; 1-B. Use GIS cluster analysis to identify areas with statistically significant clustering of high and low social vulnerability values	What patterns of relative vulnerability exist within the watershed and how do they relate to the location of hazards and amenities? Where have past watershed investments been made, and where have benefits been realized? Where do actively engaged stakeholders live and work, and how might the social conditions there create blindspots with regard to the priorities and needs of underrepresented stakeholders?
2. Temporal Vulnerability	Examination of change in indicator loadings on factors over time	How have measures of vulnerability changed over time and in relationship to where watershed investments have been made?
3. Persistent Vulnerability	Identification of geographies with stable assignment to the same vulnerability class across all decades; focus on high and low values using standard deviation from the mean	Which locations are associated with constant and high vulnerability?
4. Increasing Vulnerability	For each areal unit, examination of the slope of a line fit for the social vulnerability index over time; focus on significantly positive and negative slopes	Which areas have seen increases or decreases in social vulnerability over time? How do these relate to changing social, economic, and demographic conditions?
5. Transience as vulnerability	For each areal unit, examination of the fit (R-square) value for the line of fit describing change in vulnerability index values over time	Where is change consistent, and where is it more sporadic? Where might constant demographic transition reduce place attachment and memory?

Tools like the maps presented here can help watershed planners advance pro-environmental outcomes in ways that consider how people and places change in ways that may create new sources of vulnerability. As a result, watershed planning initiatives may better anticipate and account for the impacts of land revalorization, environmental amenity creation, and gentrification over socially vulnerable populations [18,64,78]. The analytic methods presented in this paper offer a nuanced framework for understanding the dimensions of social vulnerability and the extent that they are under consideration in watershed planning. Although better measures of vulnerability and change dynamics in and of themselves will not produce better outcomes or more holistic approaches to planning, temporal measures do broaden the potential to approach planning differently and point to prospective impacts of interventions in ways that are often never formalized within decision-making processes.

Watershed planning groups have proven to be effective allies of some of the most marginalized water resources. To become effective allies of human populations that are most underserved and marginalized, watershed planning groups can take efforts to improve their awareness of the forces shaping social vulnerability in their community.

## Supporting information

S1 Table Original variables used to construct social vulnerability measures. (DOCX)

S2 Table Structure matrix showing the loadings for all components for 1980. (DOCX)

S3 Table Structure matrix showing the loadings for all components for 1990. (DOCX)

S4 Table Structure matrix showing the loadings for all components for 2000. (DOCX)

S5 Table Structure matrix showing the loadings for all components for 2010. (DOCX)
