# Supplementary material for: Is a clean river fun for all? Recognizing social vulnerability in watershed planning
Source: PLoS One. 2018 May 1;13(5):e0196416. doi: 10.1371/journal.pone.0196416 (PMC5929536; doi:10.1371/journal.pone.0196416)
Supplement: S1 Table — (DOCX) [file pone.0196416.s001.docx]

S1 Table: Original variables used to construct social vulnerability measures and modifications made for this analysis.

| **Variable** | **Description** (Cutter 2003; Cutter et al., 2013) | **Included/Modified/Excluded** | **Effect** | **Justification (based Cutter 2003)** |
| --- | --- | --- | --- | --- |
| HODENT | Number of housing units per square mile | Included | Direct | More dense urban areas are more vulnerable (difficulties to evacuate and for emergency services to access). |
| M_C_RENT | Mean contract rent | Modified. Definition changed from mean to median contract rent to fit NCD data availability for all decades. | Inverse | Higher rent associated with higher income, so less vulnerable. |
| MHSEVAL | Mean value of owner occupied housing | Modified. Definition changed to aggregate owner-occupied home value/owner occupied housing to fit NCD data availability for all decades. | Inverse | Houses that are more expensive are less vulnerable in terms of status although houses that are more expensive represent higher financial losses. We assume more expensive houses have higher capability to deal with pollution issues and have higher insurance coverage. |
| NRREPC | Per capita residents in nursing home | Included | Direct | Limited mobility and increased reliance may increase social dependency and vulnerability to political exclusion. |
| PCTRICH | % of households earning $100,000 + | Included | Inverse | Higher earnings increase individual capacity to cope with or mitigate negative environmental impacts through access to health services, political networks, and other resources. |
| PERCAP | Per capita income (dollars) | Included | Inverse | Higher earnings increase individual capacity to cope with or mitigate negative environmental impacts through access to health services, political networks, and other resources. |
| QAGRI | % employed in farming, fishing, and forestry | Included | Direct | Dependence on local extractive resources increases direct reliance on local environmental conditions. |
| QASIAN | % Asian & Pacific Islander | Included | Direct | Ethnic and racial minority groups have traditionally been excluded and ignored in political processes. They are underrepresented among political and environmental decision makers and may perceive and experience risks differently. |
| QBLACK | % African American | Included | Direct | Ethnic and racial minority groups have traditionally been excluded and ignored in political processes. They are underrepresented among political and environmental decision makers and may perceive and experience risks differently. |

| **Variable** | **Description** (Cutter 2003; Cutter et al., 2013) | **Included/Modified/Excluded** | **Effect** | **Justification (based Cutter 2003)** |
| --- | --- | --- | --- | --- |
| QCVLBR | % of population participating in the labor force | Included | Inverse | Participating in the labor force generates income, tax revenue, and forms of empowerment that can be expressed through both labor directly and its exchange in the market. |
| QCVLUN | Unemployment | Included | Direct | Unemployment increases vulnerability by directly affecting social dependency, financial resources, emotional health, and the strength of social networks. |
| QED12LES | % of population 25+ with no high school diploma | Included | Direct | Higher levels of education are associated with lower vulnerability. The proportion of the adult population without a high school education may signal limited (scientific) literacy, lower social status, and limited employment opportunities. |
| QFEMALE | % female population | Included | Direct | Women have traditionally been excluded and ignored in political processes. They are often still underrepresented among political decision makers and may perceive and experience risks differently both because of physiological and social differences. |
| QFEMLBR | % of women participating in the labor force | Included | Inverse | Greater female labor force participation contributes to lower vulnerability through greater personal autonomy and enhanced social empowerment. |
| QFHH | Female headed families and sub-families with children | Included | Direct | More single parent (or female dependent) families are more vulnerable due to the increased social responsibility and time constraints on adults in these families. |
| QINDIAN | % Native American | Included | Direct | Ethnic and racial minority groups have traditionally been excluded and ignored in political processes. They are underrepresented among political and environmental decision makers and may perceive and experience risks differently. |
| QKIDS80 | % population > age 5 | Included | Direct | Large proportions social dependence who may also experience risk differently because of physiological differences. |
| QMOHO | % mobile homes | Included | Direct | Inexpensive and ungrounded housing is more susceptible to flooding damage, also associated with lower income groups. |
| QPOP650 | % of population age 65+ | Included | Direct | Limited mobility and increased reliance may increase social dependency and vulnerability to political exclusion. |
| QPOVTY | % of population below the poverty line | Included | Direct | Higher earnings increase individual capacity to cope with or mitigate negative environmental impacts through access to health services, political networks, and other resources. |
| **Variable** | **Description** (Cutter 2003; Cutter et al., 2013) | **Included/Modified/Excluded** | **Effect** | **Justification (based Cutter 2003)** |
| QRENTER | % renter occupied housing | Included | Direct | Renters have less direct control over the quality of the built environment and the temporal dimensions of recovery from flood and other disasters. They also may be politically marginalized because many community efforts specifically target homeowners. |
| QSPANISH80 | % Hispanic | Included | Direct | Ethnic and racial minority groups have traditionally been excluded and ignored in political processes. They are underrepresented among political and environmental decision makers and may perceive and experience risks differently. |
| QSSBEN | % households collecting social security | Included | Direct | Higher social dependency increases social vulnerability. |
| QTRAN | % employed in transportation, communication, and other public utilities | Included | Inverse | Employment in the transit industry stand in as a proxy for community mobility via public and shared-private (e.g. taxi) forms of transportation. Higher employment in this sector may be associated with greater access to modes of transport that would allow those without a personal vehicle to access political meetings and enjoy resources in other parts of the city. |
| HOSPTPC | Per capita number of community hospitals | Excluded - not available in NCD | n/a | n/a |
| MEDAGE | Median Age | Excluded - not available in NCD | n/a | n/a |
| MIGRA | % Foreign Born citizens immigrating between 1990 and 2000 | Excluded - not available in consistent form across decades in NCD | n/a | n/a |
| PPUNIT | Average number of people per household | Excluded - not available in NCD | n/a | n/a |

| **Variable** | **Description** (Cutter 2003; Cutter et al., 2013) | **Included/Modified/Excluded** | **Effect** | **Justification (based Cutter 2003)** |
| --- | --- | --- | --- | --- |
| PSYCIAN | Number of people per 100,000 employed as healthcare practitioners and technical occupations | Excluded - not available for 1980 | n/a | n/a |
| QRFRM | % rural farm population | Excluded - not available in NCD | n/a | n/a |
| QSERV | % employed in service industry | Excluded. classification changes for service industry limit comparability across decades | n/a | n/a |
| QURBN | % Urban population | Excluded - only available in NCD for 2010 | n/a | n/a |
