# Supplementary material for: Is a clean river fun for all? Recognizing social vulnerability in watershed planning
Source: PLoS One. 2018 May 1;13(5):e0196416. doi: 10.1371/journal.pone.0196416 (PMC5929536; doi:10.1371/journal.pone.0196416)
Supplement: S3 Table — (DOCX) [file pone.0196416.s003.docx]

S3 Table. Structure matrix showing the loadings for all components for 1990 (N=337). All component loadings and communalities based on a principle components analysis with varimax rotation for 25 items derived from the US Census and American Community Survey data reapportioned to the 2010 Census boundaries. Final solutions retain all components with an eigenvalue greater than one. Component loadings < |0.30| are suppressed.

| **Variable** | **C1** | **C2** | **C3** | **C4** | **C5** | **C6** |
| --- | --- | --- | --- | --- | --- | --- |
| HODENT90 | 0.31 |  | -0.44 |  | -0.51 | -0.52 |
| M_C_Rent90 | -0.40 |  | 0.74 |  |  |  |
| MHSEVAL90 | -0.43 |  | 0.94 |  |  |  |
| NRREPC90 |  | 0.54 |  |  | -0.46 |  |
| PCTRICH90 |  |  | 0.88 |  |  |  |
| PERCAP90 | -0.57 |  | 0.91 |  |  |  |
| QAGRI90 |  |  |  |  |  | 0.76 |
| QASIAN90 |  |  |  | 0.49 | -0.55 |  |
| QBLACK90 | 0.86 | 0.30 | -0.42 |  |  |  |
| QCVLBR90 | -0.38 | -0.92 | 0.31 |  |  |  |
| QCVLUN90 | 0.87 | 0.48 | -0.49 |  |  |  |
| QED12LES90 | 0.76 | 0.54 | -0.68 | 0.45 |  |  |
| QFEMALE90 |  |  |  |  | 0.52 | -0.48 |
| QFEMLBR90 | -0.32 | -0.93 |  |  |  |  |
| QFHH90 | 0.85 | 0.33 | -0.59 |  |  | -0.36 |
| QINDIAN90 |  |  |  | 0.83 |  |  |
| QKIDS90 | 0.81 |  | -0.35 | 0.37 |  |  |
| QMOHO90 |  |  |  |  |  | 0.67 |
| QPOP6590 | -0.71 | 0.46 |  |  |  |  |
| QPOVTY90 | 0.89 | 0.40 | -0.54 | 0.37 |  |  |
| QRENTER90 | 0.46 | 0.32 | -0.62 |  | -0.53 | -0.42 |
| QSERV90 | 0.82 | 0.40 | -0.63 |  |  |  |
| QSPANISH90 |  |  |  | 0.87 |  |  |
| QSSBEN90 | -0.49 |  |  |  |  |  |
| QTRAN90 | 0.80 |  | -0.72 | 0.41 |  |  |
| **Effect on social vulnerability (directionality)** | **(+)** | **(+)** | **(-)** | **(+)** | **(-)** | **(+)** |
